# Genetic polymorphisms of *MDM2 *and *TP53 *genes are associated with risk of nasopharyngeal carcinoma in a Chinese population

**DOI:** 10.1186/1471-2407-10-147

**Published:** 2010-04-18

**Authors:** Mang Xiao, Lei Zhang, Xinhua Zhu, Jun Huang, Huifen Jiang, Sunhong Hu, Yuehui Liu

**Affiliations:** 1 Department of Otorhinolaryngology-Head and Neck Surgery, Sir Run Run Shaw Hospital, Key Laboratory of Biotherapy of Zhejiang province, Zhejiang University, Hangzhou, China; 2 Department of Otolaryngology-Head and Neck Surgery, The second affected hospital to Nanchang University, Nanchang, China; 3 Department of Clinical Laboratory, Sir Run Run Shaw Hospital, Key Laboratory of Biotherapy of Zhejiang province, Zhejiang University, Hangzhou, China; 4 Department of Clinical Laboratory, Zhejiang cancer hospital, Hangzhou, China

## Abstract

**Background:**

The tumor suppressor TP53 and its negative regulator MDM2 play crucial roles in carcinogenesis. Previous case-control studies also revealed *TP53 *72Arg>Pro and *MDM2 *309T>G polymorphisms contribute to the risk of common cancers. However, the relationship between these two functional polymorphisms and nasopharyngeal carcinoma (NPC) susceptibility has not been explored.

**Methods:**

In this study, we performed a case-control study between 522 NPC patients and 722 healthy controls in a Chinese population by using PCR-RFLP.

**Results:**

We found an increased NPC risk associated with the *MDM2 *GG (odds ratio [OR] = 2.83, 95% confidence interval [CI] = 2.08-3.96) and TG (OR = 1.49, 95% CI = 1.16-2.06) genotypes. An increased risk was also associated with the *TP53 *Pro/Pro genotype (OR = 2.22, 95% CI = 1.58-3.10) compared to the Arg/Arg genotype. The gene-gene interaction of *MDM2 *and *TP53 *polymorphisms increased adult NPC risk in a more than multiplicative manner (OR for the presence of both *MDM2 *GG and *TP53 *Pro/Pro genotypes = 7.75, 95% CI = 3.53-17.58).

**Conclusion:**

The findings suggest that polymorphisms of *MDM2 *and *TP53 *genes may be genetic modifier for developing NPC.

## Background

As an important tumor suppressor, TP53 protein level is low or undetectable in normal cells, but diverse forms of stress may trigger its production, resulting in either cell cycle arrest or apoptotic cell death [1,2]. High frequencies of *TP53 *mutation and/or deletion are found in a wide variety of human malignancies, including nasopharyngeal carcinoma (NPC), which is believed to be contributed to tumorigenesis and progression [3-5].

Recently, Bond *et al*. reported that a T>G polymorphism at position 309 downstream from *MDM2 *intron 1 disrupts an Sp1 regulatory element and the *T *allele thus has a strikingly lower promoter activity compared with the *G *allele [6]. Moreover, a single nucleotide polymorphism has been identified in the coding region of *TP53*, which causes an Arg72>Pro amino acid substitution [7]. It has been shown that, compared with *Pro *allele, the *Arg *allele is faster to induce apoptosis and more efficient in suppressing transformation. Many molecular epidemiologic data found that these two polymorphisms are likely candidate genetic markers of certain cancers [8-10]. However, the gene-gene interaction of these two polymorphisms in *MDM2 *and *TP53 *has not been examined in NPC studies to date. Because of their significant impact in several tumors, these two polymorphisms might also affect the function of MDM2 and TP53 and play an important role in NPC development. These two polymorphisms might impact individual susceptibility to carcinogenesis. Based on this hypothesis, we carried out a hospital-based case-control study to investigate the relationship between polymorphisms in *MDM2 *309T>G and *TP53 *Arg72Pro and the risk of NPC in Chinese population.

## Methods

### Study Subjects

This study included 522 NPC patients and 712 healthy population controls. All subjects were ethnically homogenous Han Chinese. Patients with newly diagnosed NPC were consecutively recruited from March 2001 to May 2007, at the Sir Run Run Shaw Hospital, Zhejiang University (Hangzhou) and Zhejiang Cancer Hospital (Hangzhou). All eligible patients diagnosed at the hospital during the study period were recruited, with a response rate of 94%. Patients were from Hangzhou city and its surrounding regions and there were no age, stage, and histology restrictions. The tumor, node, metastasis (TNM) classification and tumor staging was evaluated according to the 2002 American Joint Committee on Cancer staging system [11]. The clinical features of the patients are summarized in Table 1. Population controls were cancer-free people living in Hangzhou region; they were selected from a nutritional survey conducted in the same period as the cases were collected. The control subjects were randomly selected from a database consisting of 2500 individuals based on a physical examination. The selection criteria included no history of cancer and frequency matched to cases on age and sex. Median age was 46 years (range 26-81) for case patients and 47 years (range 22-85) for control subjects (*P *= 0.78). At recruitment, informed consent was obtained from each subject. This study was approved by the Medical Ethics Committee of Sir Run Run Shaw Hospital and Zhejiang Cancer Hospital.

**Table 1:** Distribution of selected characteristics by case-control status in NPC association analysis.

	Cases		Controls	
		
	n	%	n	%
Sex				
Male	315	60.3	404	56.7
Female	207	39.7	308	42.7
Age (years)				
≤40	195	37.4	242	34.0
41-50	134	25.7	186	26.1
51-60	93	17.8	166	23.3
>60	100	19.1	118	16.6
Tumor stage				
I	126	24.2		
II	208	39.8		
III	177	33.9		
IV	11	2.1		
EBV Infection status				
Positive	329	63.1		
Negative	162	31.0		
Data missing	31	5.9		
Metastasis				
Present	283	54.2		
Absent	239	45.8		

### Polymorphism analysis

Genomic DNA was isolated from the peripheral blood lymphocytes of the study subjects. Genotypes were analyzed using PCR-based methods as described below. Genotyping was performed without knowledge of subjects' case/control status. A 30% masked, random sample of cases and controls was tested twice by different persons and the results were concordant for all masked duplicate sets.

**Figure 1:**
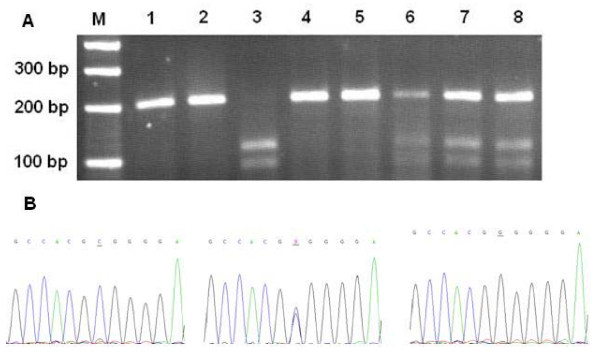
**Representative PCR-RFLP for different genotypes containing the *TP53 *Arg72Pro polymorphism site and DNA sequencing analysis**. (A) M: DNA size markers; lane 1, 2, 4 and 5: *TP53 *72Pro/Pro; lane 6, 7 and 8: *TP53 *72Arg/Pro; lane 3: *TP53 *72Arg/Arg. (B) The PCR products with different PCR-RFLP profiles were sequenced to confirm the genotypes.

The genotypes of *TP53 *Arg72Pro (rs1042522, G>C) were analyzed by PCR-RFLP method on the basis of that reported previously [12]. The primers used were *TP53 *F, 5'-TTG CCG TCC CAA GCA ATG GAT GA-3' and *TP53 *R, 5'-TCT GGG AAG GGA CAG AAG ATG AC-3', which produce a 199-bp fragment containing the G/C site. Amplification was accomplished with a 25 μl reaction mixture containing ~100 ng template DNA, 0.5 μM each primer, 0.2 mM each dNTP, 1.5 mM MgCl_2_, and 1.2 units of *Taq *DNA polymerase with 1 × Reaction buffer (Promega, Madison, WI, USA). PCR profile consisted of an initial melting step of 2 min at 94°C, followed by 35 cycles of 30 s at 94°C, 30 s at 56°C, and 30 s at 72°C, and a final elongation step of 7 min at 72°C. The 199-bp PCR products were then subject to the digestion with *BstU*I (New England Biolabs, UK) and separated on a 3.0% agarose gel. The genotypes identified by *BstU*I digestion were confirmed by DNA sequencing (Figure 1).

**Figure 2:**
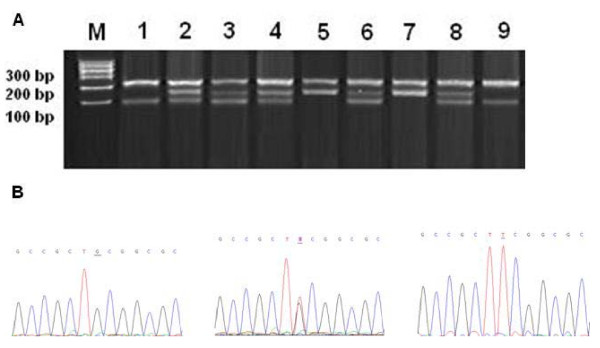
**Representative tetraprime ARMS-PCR for different genotypes containing the *MDM2 *SNP309 T>G polymorphism site and DNA sequencing analysis**. (A) M: DNA size markers; lane 1, and 9: *MDM2 *309TT; lane 2, 3, 4, 6 and 8: *MDM2 *309TG; lane 5 and 7: *MDM2 *309TT. (B) The PCR products with different tetraprime ARMS-PCR profiles were sequenced to confirm the genotypes.

*MDM2 *SNP309 (rs2279744, T>G) genotypes were analyzed using the tetra-primer amplification refractory mutation system (ARMS)-PCR method [13]. The primers for ARMS-PCR amplification of DNA fragment containing the *MDM2 *SNP309 T allele were *MDM2 *F1: 5'-GGG GGC CGG GGG CTG CGG GGC CGT TT-3' and *MDM2 *R1: 5'-TGC CCA CTG AAC CGG CCC AAT CCC GCC CAG-3'; For the *MDM2 *SNP309 G allele they were *MDM2 *F2: 5'-GGC AGT CGC CGC CAG GGA GGA GGG CGG-3' and *MDM2 *R2: 5'-ACC TGC GAT CAT CCG GAC CTC CCG CGC TGC-3'. The amplification was accomplished with a 10-μl reaction mixture containing 10 ng of template DNA, 0.8 μM of primers *MDM2 *F1 and *MDM2 *R1, 4.8 μM of primers *MDM2 *F2 and *MDM2 *R2, 0.2 mM of dNTPs, 1.5 mM of MgCl2, and 0.4 U of HotStar TaqTM with 1 × buffer and 1 × Q-solution (Qiagen, Chatsworth, CA). The reaction was carried out with an initial melting step of 15 min at 95°C, followed by 35 cycles of 45 sec at 95°C, 45 sec at 64°C, and 1 min at 72°C, and a final elongation step of 7 min at 72°C. The amplified DNA was visualized on agarose gel containing ethidium bromide. The *MDM2 *SNP309 *T *allele generated a 122-bp band, and the *G *allele generated a 158-bp band. They had a common 224-bp band, which was amplified by primers *MDM2 *F2 and *MDM2 *R1. The genotypes identified by PCR-RFLP and ARMS-PCR digestion were confirmed by DNA sequencing (Figure 2).

### Real-time analysis of *MDM2 *mRNA

Total RNA was isolated from Seventy-one NPC tissues using the Trizol reagent (Molecular Research Center, Inc., Cincinnati, OH) and converted to cDNA using an oligo (dT)_15 _primer and Superscript II (Invitrogen, Carlsbad, CA). Relative gene expression quantitation for *MDM2*, with *β-actin *as an internal reference gene, was carried out using ABI Prism 7300 sequence detection system (Applied Biosystem, Foster City, CA) in triplicates, based on the SYBR-Green method [8]. The primers used for *MDM2 *were 5'-TGT AAG TGA ACA TTC AGG TG-3' and 5'-TTC CAA TAG TCA GCT AAG GA-3'; and for *β-actin *were 5'-GGC GGC ACC ACC ATG TAC CCT-3' and 5'-AGG GGC CGG ACT CGT CAT ACT-3'. The PCR reaction mixture consisted of 0.1 μmol/L of each primer, 1 × SYBR Premix EX Taq (Perfect Real Time) premix reagent (TaKaRa, Dalian, China), and 50 ng cDNA to a final volume of 20 μL. Cycling conditions were 95°C for 10 minutes, followed by 40 cycles at 95°C of 15 seconds and 62°C for 1 minute. PCR specificity was confirmed by dissociation curve analysis and gel electrophoresis. All analysis were done in a blinded fashion with the laboratory persons unaware of genotyping data. The expression of individual *MDM2 *measurements was calculated relative to expression of *β-actin *using a modification of the method described by Lehmann *et al *[14].

### Statistical Analysis

χ^2 ^tests were used to examine the differences in the distributions of genotypes between cases and controls. The association between the *TP53 *and *MDM2 *polymorphisms and risk of NPC were estimated by ORs and their 95% CIs, which were calculated by unconditional logistic regression models. We tested the null hypotheses of multiplicative gene-gene interactions by evaluated departures from multiplicative joint effect models by including main effect variables and their product terms in the logistic regression model [15]. A more-than-additive interaction was suggested when OR_11 _> OR_10 _+ OR_01 _-1, for which OR_11 _= OR when both factors were present, OR_10 _= OR when only factor 1 was present and OR_01 _= OR when only factor 2 was present. A more-than-multiplicative interaction was suggested when OR_11 _>OR_10 _×OR_01_. The correlation of genotypes and clinical parameters was analyzed via the Fisher's exact test or χ^2 ^test as appropriate. The normalized expression values of *MDM2 *were compared by Kruskal-Wallis one way ANOVA. All *P*-values were two-sided with a *P*-value < 0.05 considered to be statistically significant. All analysis was carried out with Statistical Analysis System software (Version 9.0; SAS Institute, Cary, NC, USA).

## Results

### Allele and Genotype Distribution

The genotype results are shown in Table 2. The allele frequencies for *MDM2 G *and *TP53 Pro *were 0.423 and 0.426 in controls, and 0.555 and 0.518 in cases respectively. The observed genotype frequencies of *MDM2 *and *TP53 *polymorphisms in both controls and cases did not deviated from those expected from the Hardy-Weinberg equilibrium. Distributions of these *MDM2 *and *TP53 *genotype were then compared among cases and controls. The frequencies of *MDM2 *TT, TG and GG genotypes among patients were significantly different compared to controls (*P*_*trend *_< 0.001), with the GG homozygotes being significantly overrepresented among patients compared to controls (*P *< 0.001). Moreover, Logistic regression analysis showed that subjects with *TP53 Pro *allele significant increased risk of NPC compared with subjects carrying the *Arg *allele (OR for the Arg/Pro genotype, 1.43; 95%CI, 1.22-2.13; OR for the Pro/Pro genotype, 2.22; 95%CI, 1.58-3.10; *P*_*trend *_< 0.001), suggesting that the *Pro *allele is the high-risk allele.

**Table 2:** Genotype and allele frequencies of *TP53 *and *MDM2 *among cases and controls and their association with the risk of NPC.

Genotype	Cases (n = 522)	Controls (n = 712)	OR* (95% CI)
			
	no. (%)	no. (%)	
*TP53 *72Arg>Pro			
Arg/Arg	117 (22.4)	226 (31.7)	1.00 (reference)
Arg/Pro	270 (51.7)	366 (51.4)	1.43 (1.22-2.13)
Pro/Pro	135 (25.9)	120 (16.9)	2.22 (1.58-3.10)
*Pro *allele frequency	0.518	0.426	
*MDM2 *309T>G			
TT	111 (21.2)	238 (33.4)	1.00 (reference)
TG	243 (46.6)	346 (48.6)	1.49 (1.16-2.06)
GG	168 (32.2)	128 (18.0)	2.83 (2.08-3.96)
*G *allele frequency	0.555	0.423	

*OR and 95%CI were calculated by unconditional logistic regression, with *TP53 *Arg/Arg or *MDM2 *TT genotype as the reference group and adjusted by sex and age.

The effects of the *TP53 *and *MDM2 *polymorphisms were additionally examined with stratification by age, tumor size, metastatic status and Epstein-Barr virus (EBV) infection status. However, no significant association was observed between age and the TNM stage at the time of NPC diagnosis and the polymorphism of this gene, and no interaction was detected between the polymorphism and status of EB virus infection (data not shown).

### Gene-Gene Interaction between *MDM2 *and *TP53 *Polymorphisms

We examined whether there was a statistical interaction between the *MDM2 *and *TP53 *polymorphisms (Table 3). The data showed that patients who carried the *MDM2 *GG genotype were also more likely to carry the *TP53 *Pro/Pro genotype than the controls (10.3% vs. 2.8%, *P *< 0.001). The presence of one *MDM2 *GG genotype, but not one *TP53 *Pro/Pro genotype, were associated with an increased risk of NPC (OR = 2.83, 95% CI = 1.33-5.90), compared to the lack of such a genotype. However, the presence of both *MDM2 *GG and *TP53 *Pro/Pro genotypes was associated with an even higher risk for NPC increase (OR = 7.75, 95% CI = 3.53-17.58; *P *< 0.05, test for homogeneity) compared to those who lacked both genotypes. These results clearly indicate a more than multiplicative interaction [15] between the *MDM2 *GG and *TP53 *Pro/Pro genotype in the risk of developing NPC.

**Table 3:** Risk of NPC associated with *MDM2 *genotypes by *TP53 *genotypes.

Genotype		Cases	Controls	OR* (95% CI)
			
*MDM2 *309T>G	*TP53 *72Arg>Pro	no. (%)	no. (%)	
TT	Arg/Arg	24 (4.6)	68 (9.6)	1.00 (reference)
	Arg/Pro	63 (12.1)	128 (18.0)	1.34 (0.75-2.48)
	Pro/Pro	24 (4.6)	42 (5.9)	1.63 (0.81-3.42)
TG	Arg/Arg	54 (10.3)	120 (16.9)	1.30 (0.72-2.39)
	Arg/Pro	132 (25.3)	168 (23.6)	2.12 (1.90-3.86)
	Pro/Pro	57 (10.9)	58 (8.1)	2.91 (1.53-5.31)
GG	Arg/Arg	39 (7.5)	38 (5.3)	2.83 (1.33-5.90)
	Arg/Pro	75 (14.4)	70 (9.8)	3.13 (1.61-5.94)
	Pro/Pro	54 (10.3)	20 (2.8)	7.75 (3.53-17.58)

* Data were calculated by unconditional logistic regression, adjusting for sex and age status.

### *MDM2 *RNA Levels in NPC tissues from Different Genotype Carriers

**Figure 3:**
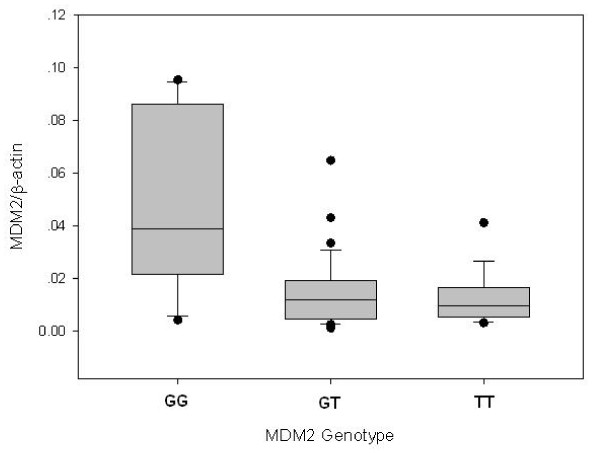
***MDM2 *mRNA expression level in NPC tissue as function of *MDM2 *genotype**. Columns, mean; bars, ± SD normalized to *β-actin*. Expression level among the GG genotype was significantly different from that among the GT or TT genotype (P < 0.05).

To examine the effect of the *MDM2 *309T>G polymorphism on MDM2 expression in the target tissues, the levels of *MDM2 *mRNA in individual NPC tissues were quantified by real-time PCR (Figure 3). The results showed that the *MDM2 *GG genotype carriers (n = 19) had significantly higher *MDM2 *mRNA level than the *MDM2 *TT genotype carriers [0.050 ± 0.033 (n = 19) versus 0.014 ± 0.009 (n = 18), *P *< 0.001]. However, the *MDM2 *TG genotype carriers (n = 34) had a *MDM2 *mRNA level that was very similar to that of the TT genotype carriers (0.015 ± 0.013 versus 0.014 ± 0.009, *P *= 0.610).

## Discussion

In the present study, our group found that *MDM2 *and *TP53 *polymorphisms may influence the development of NPC in a Chinese population. On the basis of examining 522 cases and 712 controls, our data showed that *MDM2 *309GG, which increase *MDM2 *expression level in NPC tissue, and *TP53 *72Pro/Pro genotypes were statistically significantly associated with increased risk of NPC. In addition, the association between these two polymorphisms and the risk of NPC displayed a multiplicative gene-gene interaction, which rendered the subjects carrying both *MDM2 *309GG and *TP53 *72Pro/Pro genotypes at much higher risk for developing NPC.

Our results showing an association between risk of NPC and polymorphisms of *MDM2 *and *TP53 *are biologically plausible for the following reasons. Firstly, there is broad evidence suggesting that *TP53 *is a key gene in maintaining genomic integrity and preventing tumorigenesis [16-19]. The association between mutation of *TP53 *and susceptibility to tumor formations has been tested in several studies with genetically modified animals. It was found that mice lacking the inactivating mutation in one *tp53 *allele developed fewer tumors than mice harboring it and they developed tumors very early in life and at very high frequencies [20]. Moreover, overexpression of *MDM2*, which can led to loss of *TP53 *activity, was also observed in a variety of tumors with diverse tissue origins [21,22]. Secondly, the investigated polymorphisms in the *TP53 *and *MDM2 *genes have functional consequences [6,7,23]. Our real-time PCR finding is consistent with recent reports by Bond *et al*. and Hong *et al *[6,8] that the *MDM2 *309GG genotype carriers had significantly higher *MDM2 *expression in NPC tissues than the TT and TG genotype carriers, suggesting the variant *MDM2 *genotype may cause attenuated TP53 function.

Several case-control studies have examined the association between these two polymorphisms and many tumor types, but the results are conflicting [8,9,24-27]. A meta-analysis of 21 studies showed that ORs of a variety of cancers associated with the *MDM2 *GG and TG genotype were 1.17 (95% CI = 1.04-1.33) and 1.15 (95% CI = 1.03-1.28), respectively [28]. Moreover, another meta-analysis study reported that the *TP53 *Pro/Pro polymorphisms was significantly increase susceptibility to NPC [29]. Phang *et al*. reported in a study conducted in Singapore Chinese that the *MDM2 *309SNP was not associated with leukemia [30]. However, Xiong *et al*. also found an increased risk of acute myeloid leukemia (AML) associated with *MDM2 *309GG genotype [9]. Zhou *et al*. shown that MDM2 309SNP may be a risk factor for occurrence of NPC [31]. Moreover, the control frequencies of *TP53 *Arg72Pro and *MDM2 *SNP309 in our present study were similar with Asian population in published papers [28,29]. Our study provided strong molecular epidemiologic evidence to support the hypothesis that *TP53 *72Arg/Pro and *MDM2 *309T>G polymorphisms also affect the development of NPC.

Although it is generally believed that TP53 pathway also plays a critical role in tumor aggressive course [32,33], we did not find significant correlations between *TP53 *and *MDM2 *genotypes and the prognosis status of NPC in the present study. These results suggest that the examined polymorphisms in *TP53 *and *MDM2 *might not serve as a sole risk marker of prognosis. Further examinations of larger patient series with prospectively follow-up clinical outcomes especially the survival rates may be required. Moreover, our study may have certain limitations because of the study design. Selection bias and/or systematic error may occur because the cases were from the hospital and the controls were from the community. Some factors which may interact with genotype or act as potential confounders in analysis such as information of nutrition is not available in our case-control study.

## Conclusion

The current study demonstrated a significant association between the *TP53 *72Arg/Pro and *MDM2 *309T>G polymorphisms and the risk of developing NPC for the first time. The association of *MDM2 *polymorphism with the risk of NPC displayed a multiplicative gene-gene interaction with the *TP53 *72Arg/Pro polymorphism. These molecular epidemiology findings are consistent with the results obtained from the functional analysis. Because *MDM2 *overexpression and high frequencies of *TP53 *mutation are found in many tumor types, additional studies on other tumor types would be warranted. Moreover, the possible role of these polymorphisms in disease prognosis should also be addressed in the future studies.

## Abbreviations

*MDM2*: mouse double minute 2; CI: confidence interval; OR: odds ratio; NPC: nasopharyngeal carcinoma; SNP: single nucleotide polymorphism.

## Conflicts of interest statement

The authors declare that they have no competing interests.

## Authors' contributions

MX participated in molecular genetic studies and drafted the manuscript. LZ carried out bioinformatics analysis and critically revised the manuscript. XZ performed the genotyping and statistical analysis and participated in the critical revision of the manuscript. JH, HJ and SH participated in collection of data and manuscript preparation. YL conceived of the study, and participated in its design and coordination. All authors read and approved the final manuscript.

## Pre-publication history

The pre-publication history for this paper can be accessed here:

http://www.biomedcentral.com/1471-2407/10/147/prepub
